# Functional Studies of p.R132C, p.R149C, p.M283V, p.E431K, and a Novel c.652-2A>G Mutations of the *CYP21A2* Gene

**DOI:** 10.1371/journal.pone.0092181

**Published:** 2014-03-25

**Authors:** Melisa Taboas, Luciana Gómez Acuña, María Florencia Scaia, Carlos D. Bruque, Noemí Buzzalino, Mirta Stivel, Nora R. Ceballos, Liliana Dain

**Affiliations:** 1 Centro Nacional de Genética Médica, Administración Nacional de Laboratorios e Institutos de Salud (ANLIS), Dr. Carlos G. Malbrán, Buenos Aires, Argentina; 2 Instituto de Biología y Medicina Experimental, Consejo Nacional de Investigaciones Científicas y Técnicas (IBYME-CONICET), Buenos Aires, Argentina; 3 Instituto de Fisiología, Biología Molecular y Neurociencias (IFIBYNE), Facultad de Ciencias Exactas y Naturales, Universidad de Buenos Aires y Consejo Nacional de Investigaciones Científicas y Técnicas (CONICET), Buenos Aires, Argentina; 4 Departamento de Biodiversidad y Biología Experimental, Facultad de Ciencias Exactas y Naturales, Universidad de Buenos Aires y Consejo Nacional de Investigaciones Científicas y Técnicas (CONICET), Buenos Aires, Argentina; 5 División Endocrinología, Hospital Durand, Buenos Aires, Argentina; Instituto de Ciencia de Materiales de Madrid - Instituto de Biomedicina de Valencia, Spain

## Abstract

Congenital adrenal hyperplasia (CAH) due to 21-hydroxylase deficiency is the most frequent inborn error of metabolism and accounts for 90–95% of CAH cases. In the present work, we analyzed the functional consequence of four novel previously reported point *CYP21A2* mutations -p.R132C, p.R149C, p.M283V, p.E431K- found in Argentinean 21-hydroxylase deficient patients. In addition, we report an acceptor splice site novel point mutation, c.652-2A>G, found in a classical patient in compound heterozygosity with the rare p.R483Q mutation. We performed bioinformatic and functional assays to evaluate the biological implication of the novel mutation. Our analyses revealed that the residual enzymatic activity of the isolated mutants coding for CYP21A2 aminoacidic substitutions was reduced to a lesser than 50% of the wild type with both progesterone and 17-OH progesterone as substrates. Accordingly, all the variants would predict mild non-classical alleles. In one non-classical patient, the p.E431K mutation was found in *cis* with the p.D322G one. The highest decrease in enzyme activity was obtained when both mutations were assayed in the same construction, with a residual activity most likely related to the simple virilizing form of the disease. For the c.652-2A>G mutation, bioinformatic tools predicted the putative use of two different cryptic splicing sites. Nevertheless, functional analyses revealed the use of only one cryptic splice acceptor site located within exon 6, leading to the appearance of an mRNA with a 16 nt deletion. A severe allele is strongly suggested due to the presence of a premature stop codon in the protein only 12 nt downstream.

## Introduction

Congenital adrenal hyperplasia (CAH) due to 21-hydroxylase deficiency (OMIM 201910) accounts for 90–95% of CAH cases [1], [2]. This autosomal recessive disorder, which is the most frequent inborn error of metabolism, has a broad spectrum of clinical forms, ranging from severe or classical (CCAH) to the mild late onset or non-classical one (NCCAH) [1]. The classical form includes salt-wasting (SW) and simple virilizing (SV) variants, depending on the degree of aldosterone deficiency. Girls with classical CAH are typically born with ambiguous genitalia. Patients with NCCAH exhibit clinical manifestations of hyperandrogenism.

Neonatal screening programs performed since 1977 have shown an overall incidence of 1∶15000 live births for the classical form [3]–[6]. On the other hand, NCCAH is estimated to be more common than CCAH, with a prevalence of 1∶1000 in the white population, and more frequent in certain ethnic groups such as Jews of Eastern Europe, Hispanics and Yugoslavs [7].

The affected enzyme, CYP21A2, is encoded by the *CYP21A2* gene. This gene is located in 6p21.3 adjacent to a pseudogene, *CYP21A1P*, with which it shares 98% of nucleotide sequence identity. Due to the high degree of identity between *CYP21A2* gene and its pseudogene, most of the disease-causing mutations are likely the consequence of non-homologous recombination or gene conversion events [8], [9]. Even though most patients carry *CYP21A1P*-derived mutations, an increasing number of naturally occurring mutations have been found in disease-causing alleles in the last years (see: http://www.hgmd.cf.ac.uk for details). Mutations in the *CYP21A2* gene cause varying degrees of 21-hydroxylase activity loss [10]. *In vitro* studies have revealed that usually mutations leading to a complete inactivation of 21-hydroxylase activity are associated with the SW phenotype. Those that reduce enzyme activity close to 2% cause the SV phenotype, while those with a remaining enzymatic activity in the range of 10% to 70% result in the mild NCCAH phenotype.

Recently, efforts have been made towards predicting activities of mutant proteins and homology modeling has emerged as a useful tool to evaluate, through structure-based methods, protein activity or stability impairment. Although the use of approximate models allows making some predictions with certain accuracy [11]–[13], *in vitro* studies should be conducted to further analyze the putative biological implication of the mutant alleles. Moreover, the severity of the impairment of enzymatic activity of two mild mutations on the same allele could be difficult to predict in the absence of functional studies, studies being essential for genetic counselling as this allele could represent a severe one.

In this paper, we analyzed the functional consequence of four previously reported novel point *CYP21A2* mutations leading to aminoacidic substitutions, p.R132C, p.R149C, p.M283V, p.E431K, found in Argentine patients [12]. For these mutations, *in silico* molecular modeling analyses have revealed changes in the stability and/or surface charge of the protein that could be related to the clinical manifestations found in the patients [12].

In addition, we report a novel point mutation, c.652-2A>G, in the intron 5 acceptor splicing site found in a SV patient from our cohort in compound heterozygosity with the rare p.R483Q mutation. Its pathogenic functional implication was also analyzed by means of bioinformatic predictions and functional assays.

## Materials and Methods

All clinical investigations were conducted according to the principles expressed in the Declaration of Helsinki and written informed consent was obtained from the patient. The study was approved by the Ethic Committee of the Instituto de Biología y Medicina Experimental, Buenos Aires, Argentina.

### Patients

Clinical symptoms of the patients in whom the four aminoacidic mutations were found have been described previously [12].

Physical and endocrine evaluation of the patient in whom the novel mutation in the intron 5 acceptor splicing site was found, was conducted at the Division Endocrinología of the Hospital Durand, Buenos Aires, Argentina. Clinical characteristics and hormonal profile of this SV patient are presented in Text S1.

### Materials

Primers were from Sigma-Aldrich (St. Louis, MA). Fugene HD Transfection, β-galactosidase pSV-Gal vector, lysis buffer and β-Galactosidase Enzyme Assay System were obtained in Promega (Madison, WI). PureLink, Quick Plasmid Miniprep Kit, DMEM medium High Glucose and Pyruvate, Lipofectamine, and MMLV-RT reverse transcriptase were purchased in Invitrogen (Carlsbad, CA). Wild-type plasmid pCMV4-CYP21A2 was kindly provided by Dr. A. Wedell (Karolinska Institute, Sweden) and antihuman CYP21A2 rabbit polyclonal antiserum was kindly provided by Dr. W. L. Miller (University of San Francisco, San Francisco, CA). QuikChange multi-site-directed mutagenesis kit was obtained in Stratagene (La Jolla, CA). Radioactive substrates were from Perkin-Elmer (Boston, MA). Rabbit anti β-actin antibody was purchased in Santa Cruz Biotechnology (Santa Cruz, CA). QIAquick Gel Extraction Kit was from Qiagen (Hilden, Germany) and Tris Reagent was from Molecular Research Center, Inc. (Cincinnati, OH). Silica gel plates 60GF 254 on aluminum were purchased from Merck (Darmstadt, Germany). Scintillation counting was carried out with Wallac 1409 DSA equipment with OptiPhase-Hi safe 3 as scintillation cocktail (Wallac Co, Turku, Finland).

### DNA Analyses

Nucleotide numbering was performed following the guidelines of the Human Genome Variation Society [14], using M13936.1 [15] as the genomic *CYP21A2* reference sequence and NM_000500.7 NCBI Reference Sequence for mRNA. All new data has been deposited in GeneBank database.

Screening of the 10 most frequent mutations in the *CYP21A2* gene in DNA sample from the CCAH patient was performed as previously described [16], [17]. Since none of these points of mutations were found, DNA was further analyzed by direct sequencing [12]. Parents were not available, thus the presence of mutations in *trans* was assumed. In addition, DNA samples of 50 randomly selected subjects from the general population were screened for the novel mutation. Briefly, a *CYP21A2* specific fragment from exon 1 to exon 6 was amplified with primers already described [18], [19]. Screening of the g.1329A>G (c.652-2A>G, Genbank KF051996) mutation was performed in a second round of PCR by amplification of a 696-bp fragment spanning exon 3 to 6 [12], followed by a BtgI restriction enzyme assay and a 2% Ethidium Bromide-stained agarose gel electrophoresis. Fragments from wild type allele were of 84 and 612 bp, while the mutant allele rendered 75, 84 and 537 bp fragments.

### Bioinformatic Analysis

*In silico* analysis was performed to assess the disrupted effect of c.652-2A>G mutation and the presence of putative cryptic acceptor sites. This search was performed using The Human Splicing Finder tool (HSF, http://www.umd.be/HSF/) [20]. A fragment of 206 pb ranging from nt 1221 in the 3′ end of exon 5 to nt 1425 in the exon 6 was analyzed. For the examination of putative cryptic sites, a threshold of 80% in the consensus value (CV) was considered, as it represents a value for strong splicing sites. Likewise, a reduction in the CV (ΔCV) of at least 10% for a mutation is likely to have a significant impact on splicing and should be further investigated [20].

### *In vitro* Expression and Assay of 21-hydroxylase Activity

*In vitro* studies were carried out for the aminoacidic mutations as described by Krone *et al.* [21] with slightly modifications. Since the p.E431K mutation was found in *cis* with another described point mutation (p.D322G) [22], all the variants were tested in isolation and the double mutant was also analyzed. The p.I172N with about 2% residual activity and responsible for SV form [23] was used as a low-activity positive control. Mutagenesis of the wild-type (WT) pCMV4-*CYP21A2* plasmid was performed with the QuikChange multi-site-directed mutagenesis kit according to the manufacturer’s instructions. At least two different colonies for each mutation were collected, and the plasmids verified by sequencing the whole insert. The tested plasmids were: WT- pCMV4-*CYP21A2* for reference, pCMV4 without *CYP21A2*cDNA as negative control, and mutated pCMV4-*CYP21A2*.

Approximately 1.6×10^5^ COS-7 cells (ATCC CRL-1651) were transiently transfected by Fugene HD Transfection with 1 μg each of pCMV4-*CYP21A2* construct and β-galactosidase pSV-Gal vector in 6 well plate and incubated in DMEM-F12 medium supplemented with 10% fetal bovine serum (FBS) for 48 h. COS-7 line was used up to 10 passages. The activity of 21-hydroxylase in intact COS-7 cells was determined after transfection by measuring the conversion of [^3^H]progesterone and [^3^H]17-hydroxyprogesterone (17-OHP) into [^3^H]deoxycorticosterone and [^3^H]11-deoxycortisol, respectively. Cells were incubated with 0.5 μCi of each of the radioactive substrates and 2.0 μM unlabeled steroids with or without 5 mM NADPH, 1h at 37°C. Media were collected and steroids analyzed by thin layer chromatography (TLC) as previously described [24]. Substrate conversion rates for mutant CYP21A2 were compared with those of WT enzyme. Cells were washed with PBS and 400 μl lysis buffer was added. Plates were incubated during 15 min on ice-water bath and subsequently homogenized in the same buffer. Homogenates were centrifuged and the supernatant transferred to clean tubes. Proteins in the lysate were quantified by Bradford assay [25], and β-galactosidase activity was measured by β-Galactosidase Enzyme Assay System according to manufacturer’s instructions. The activity of mock transfection was deducted in each experiment. Enzyme activities of the mutants were expressed as percentage of WT CYP21A2-activity that was defined as 100%. Each experiment was performed two to four times and the ratio of β-galactosidase activity to total protein content was measured in each experiment to verify the reproducibility of transfection.

Expression of the WT and mutant proteins was studied by Western blotting. A total amount of 23 μg of protein was electrophoresed in 10% SDS-PAGE gel. After transference, membranes were incubated for 10 min in PBS-0.1% Tween-20 (PBS-T) and 3% hydrogen peroxide and blocked for 1 hr at room temperature in PBS-T containing low-fat powdered milk (5%). Incubations with primary antibodies were performed over night at 4°C with 1/30000 antihuman CYP21A2 rabbit polyclonal antiserum [26] or 1/800 mouse anti β-actin antibody. Secondary antibodies were anti-mouse and anti-rabbit conjugated to peroxidase. Immunopositive bands were detected using 1.25 mM luminol, 0.198 mM cumaric acid and 0.038% v/v hydrogen peroxide in 100 mM Tris-HCl buffer. Densitometric analysis of the corresponding bands was performed with the ImageGauge software (Fuji photo film CO., LTD, Altura Software Inc). At least two different extracts were analyzed for each mutant and each of them was processed twice. Protein levels of the different *CYP21A2* constructs were expressed as the ratio between CYP21A2 and β-actin, considering β-galactosidase activity as the control for transfection efficiency. For quantification, the background expression of the mock construct was deducted.

### Minigene Construction, Transfection and mRNA Analysis

A minigene with a fragment spanning from exon 4 to exon 7 was constructed. After the first amplification to select *CYP21A2* gene [16], a nested-PCR was performed to construct the minigene with the following primers: exon 4 sense-BstEII 5′- GCGGGTGACCCatcatctgttacctcaccttc-3′ and exon 7 antisense-BsteII 5′-gccggtCaccGtttgctgtggtctcagtg-3′. PCR product was digested with *BstEII* and ligated into a *BstEII* digested pSVEDEDBXho vector [27]. Clones were sequenced to verify their identity. Recombinant plasmids were purified by PureLink, Quick Plasmid Miniprep Kit, and transfected in HEK-293 (ATCC CRL-1573), HeLa (ATCC CCL-2) and Y-1 cells (ATCC CCL-79). For HEK-293 and HeLa cells, approximately 2×10^5^ cells were plated in a 6 well plate containing DMEM medium high glucose with or without pyruvate, respectively, and supplemented with 10% FBS and. After 24 hours, HEK-293 and HeLa cell cultures were transfected with 2 μg of total DNA (100 ng of minigene and the rest pBlueScript used as carrier DNA) using Lipofectamine 6 μl/well. For Y-1 cells, approximately 5×10^4^ cells were plated in a 24 well plate containing HAM-F10 medium supplemented with 2.5% FBS, 12.5% horse serum. After 24 hours Y-1 cells cultures were transfected with Fugene HD (2 μl/μg of DNA). All cell lines were used up to 10 passages.

Cells were collected 48 hours after transfection and total RNA was isolated by using Tris Reagent. First-strand synthesis was performed using MMLV-RT reverse transcriptase. The cDNA was amplified by PCR using the following primers spanning the pSVEDEDBXho vector: sense 5′ACTGCCTGCTGGTGACCCATC3′ and antisense 5′CGGCCAGGGTCACCGTTTG3′ in the presence of 0.5 μCi of ^hello^α^32^-P]dCTP, resolved in a 6% acrylamide-bisacrylamide (29∶1) gel and revealed by autoradiography. An aliquot of a non-radioactive PCR was further analyzed by direct sequencing.

## Results

### Novel Mutation

**Figure 1:**
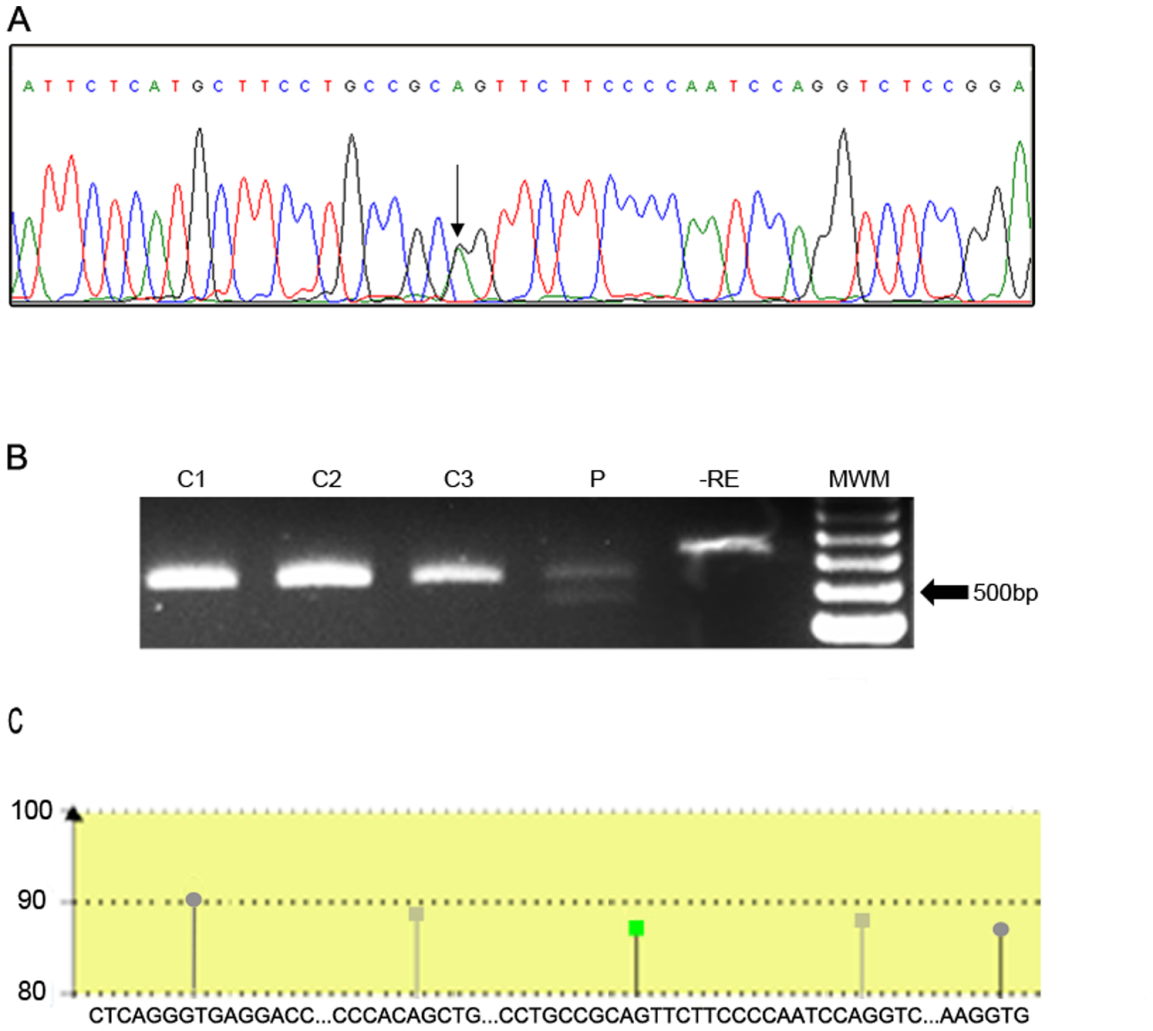
Representative electropherogram and restriction enzyme assay of the novel splice mutation found. A: Mutation g.1329A>G (c.652-2A>G) changes an acceptor splice site in intron 5. The change A>G is depicted by an arrow. B: BtgI restriction enzyme assay for c.652-2A>G. C: control individuals, P: patient. -RE: control without restriction enzyme. MWM: molecular weight marker. C: Cartoon representation of the bioinformatic results. A fragment of 206 pb ranging from nt 1221 in the 3′ end of exon 5 to nt 1425 in the exon 6 was analyzed using the Human Splicing Finder tool. Only the main sites were included in the diagram. Green square: consensus acceptor site in the intron 5 disrupted by the mutation; grey squares: putative critic acceptor sites located 5′and 3′ of the canonical one; grey circles: consensus donor sites.

Five novel disease-causing mutations were detected in patients from our cohort. Four out of five were associated with NCCAH patients: p.R132C, p.R149C, p.M283V and p.E431K, and were previously described [12]. One of the mutations, g.1329A>G, now named c.652-2A>G, is herein described for the first time (Figure 1A) and was found in a SV patient. The g.1329A>G mutation was not detected in 100 chromosomes of randomly selected individuals of the general population (1B).

The intronic mutation found at position 1329, being A changed to G, is coincident with the 3′ splice site of intron 5. To evaluate the putative use of cryptic splicing sites by the novel mutation, we used the human splicing finder (HSF) V.2.4.1.tool [20]. An inactive site with a difference in the consensus value (ΔCV) of −32,5% associated to c.652-2A>G mutation and two strong cryptic splice sites with a CV higher than 80% (one in the middle of intron 5 and one in exon 6) were predicted (Figure 1C).

### Functional Assays

#### Enzyme activity and Western Blot analysis

To assess the influence of the different base substitutions in the coding region of 21-hydroxylase, the activity of the normal protein with those of the various mutant forms were compared. All mutant forms of CYP21A2 were transiently expressed in COS-7 cells [21], and enzyme activities in intact cells assayed by using the two natural substrates for 21-hydroxylase, progesterone, and 17-OHP.

**Figure 2:**
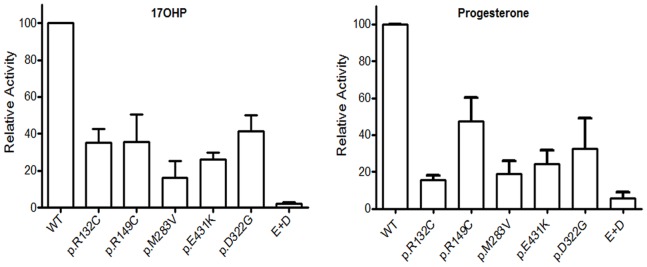
Residual activity of the different mutants. Enzyme activities were expressed as percentage of wild type CYP21A2 activity that was defined as 100%. Conversion values are shown for the two natural substrates (17-OHP to 11-deoxycortisol and progesterone to 11-deoxycorticosterone). Each bar represents the mean ± S.E.M. of two different colonies for each mutant assayed 2 to 4 times in independent experiments.

Figure 2 shows the percentage of the remaining activity of all aminoacidic mutants of CYP21A2 in comparison with the activity of the WT that was arbitrarily defined as 100%. As shown, for both substrates, the enzymatic residual activity for all the isolated mutations was lower than 50%.

Considering that the mutations p.E431K and p.D322G were found in the same allele in one NCCAH patient, the combined effect of the two missense mutations was also analyzed. Both mutations were synergistic, resulting in a reduction of the activity near to a 2% and 5% of the WT activity for 17-OHP and progesterone, respectively. Table 1 summarizes the results of the *in vitro* enzymatic activity found for the alleles with the novel mutations found in these patients. Isolated p.E431K and p.D322G mutations have residual enzymatic activities of 26.2±3.8% and 41.6±8.4%, respectively, when 17-OHP was assayed as a substrate, and 24.2±7.4% and 32.4±16.7%, respectively, for progesterone. As a control, p.I172N mutation associated with the classical form of the disease was used. In our conditions, p.I172N residual activity was 1.22±0.13%. The presence of NADPH in the culture media had no effect on enzyme activity (data not shown).

**Table 1:** *In vitro* residual enzymatic activity of the allele bearing the novel mutation found for the patients.

Patient	Phenotype	Genotype	Mean residual activity of the allele with the novel mutation (SEM)	
			17-OHP	Progesterone
**1**	NCCAH?	**p.R132C**/N	35.4 (7.4)	15.5 (2.7)
**2**	NCCAH	**p.R149C**/p.V281L	35.8 (14.6)	47.3 (12.9)
**3**	NCCAH	**p.M283V**/c.283-13A/C>G	16.2 (9.3)	19.0 (6.8)
**4**	NCCAH	p.D322G-**p.E431K**/p.V281L	2.1 (1.1)	5.6 (3.3)

NCCAH: non.classical CAH. 17-OHP: 17-OH progesterone. Novel mutations are in bold and underlined. Residual activity is expressed as percentage relative to the WT allele that was considered as 100%.

**Figure 3:**
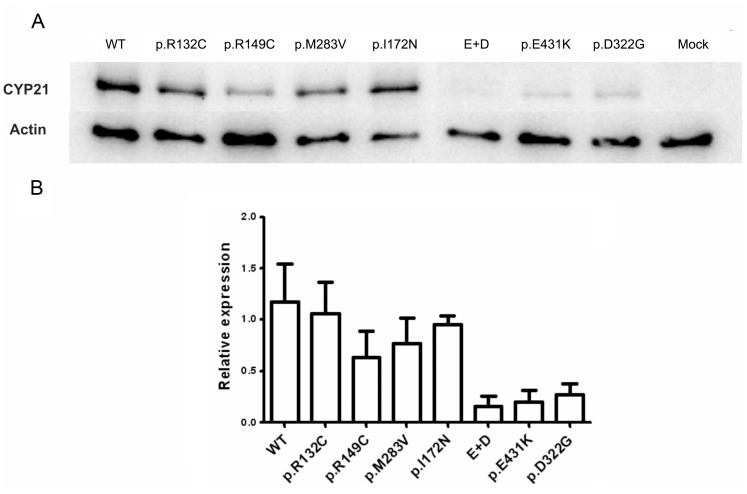
Expression analysis of the different mutants. A: Representative western blot showing expression levels of wild type CYP21 and the different mutagenized constructions assayed. B: Densitometric quantification of CYP21A2 expression. Protein levels of the different CYP21A2 constructs were expressed as the ratio between CYP21A2 and β-actin, considering β-galactosidase activity as the control for transfection efficiency. Bars represent the mean ± S.E.M of 2 different extracts assayed 2 times each.

To investigate whether the mutations in *CYP21A2* affect the level of the protein expression, western blot studies were performed with proteins obtained from homogenates of cells transfected with the different constructs. Figure 3 shows a representative western blot analysis. Of note, p.E431K, p.D322G and p.E431K+ p.D322G mutations clearly showed a lower amount of CYP21A2.

#### Minigene and mRNA analysis

**Figure 4:**
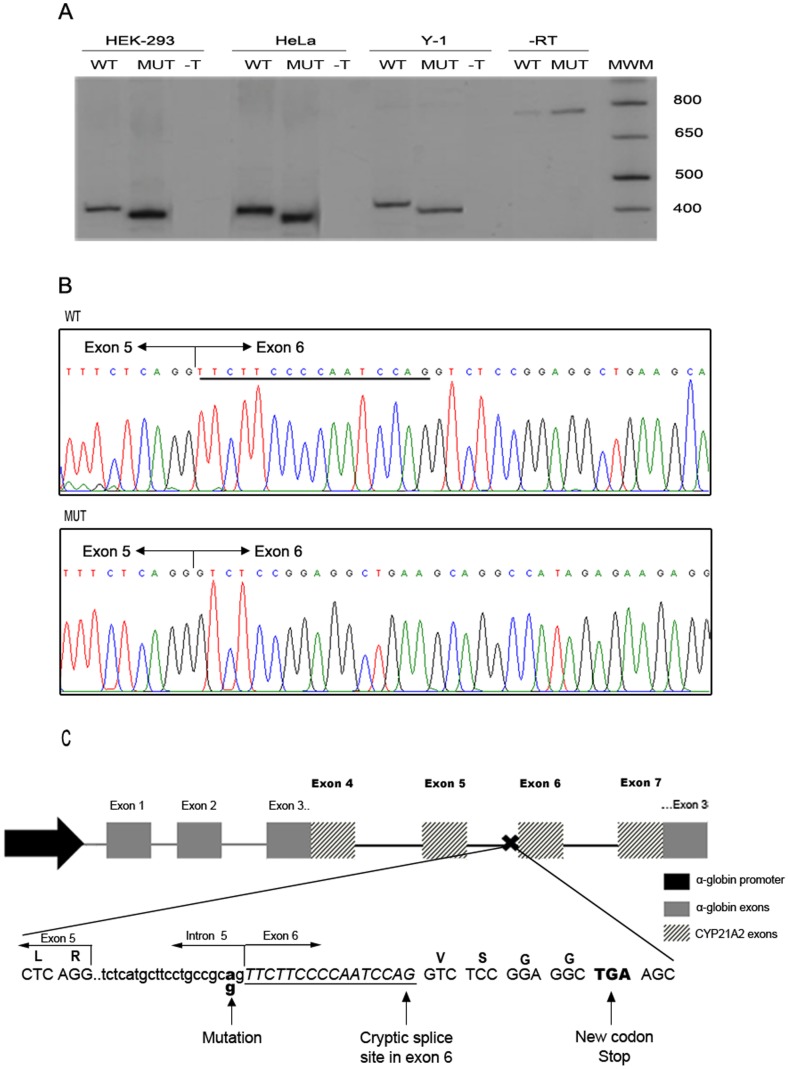
pre-mRNA splicing analysis of wild type (WT) and mutant (MUT) c.652-2A>G *CYP21A2* genes. A: Representative polyacrilamide gel electrophoresis of a Reverse Transcription (RT)- PCR analysis of *CYP21A2* minigene mRNAs in 3 cells types: HEK-293, HeLa and Y-1. –T: not transfected. -RT: without reverse transcription: bands observed in both lanes correspond to PCR products from plasmid DNA used as template. MWM: Molecular weight marker. B: Representative electropherograms of the RT-PCR products. Junction of exon 5 and 6 are depicted in each case. Underlined, the 16 nt present in the wild type sequence and absent in the mutant one. C: Diagram summarizing the strategy used and the results obtained. Letters above the sequence represent the codified aminoacids. The 16 nt deletion is in italic and underlined. Dashed arrows represent primers used in the RT-PCR spanning the α-globin and *CYP21A2* regions of the minigene.

After the bioinformatics results, we analyze the splicing outcome of the gene bearing the novel c.652-2A>G mutation. Splicing reporter minigenes were constructed comprising the genomic region from exon 4 to exon 7 of the *CYP21A2* gene, wild type or mutated, inserted in exon 3 of the *α-globin* gene (Figure 4C). The vector carrying this construct, under the control of the α-globin promoter, was transiently transfected into two different human cells lines, HEK-293 and HeLa, and in the murine steroidogenic Y-1 cells. After RNA extraction, splicing was assessed by RT-PCR using primers that map the junction between the *α-globin* and the *CYP21A2* regions of the minigene, thus amplifying only the mRNA product of the transfected minigenes. As a result, we observed that both the mutated and the WT constructs lead to only one mRNA isoform each but of different lengths, being the mutant shorter that the WT (Figure 4A). Sequencing of this PCR products revealed that the allele with the c.652-2A>G mutation completely abolishes the use of the canonical splice site. Instead, the use of the cryptic splice acceptor site within exon 6 created an mRNA with a 16 nt deletion (Figure 4B). As a consequence, the appearance of a premature stop codon is predicted 12 nt downstream (Figure 4C).

## Discussion

The adrenocortical 21-hydroxylase is one of the key enzymes in glucocorticoid and mineralocorticoid biosynthesis. Mutations in the *CYP21A2* gene are responsible of CAH due to 21-hydroxylase deficiency. To date, a great number of different mutations in *CYP21A2* have been described. The majority of these mutations result in amino acid substitutions that may disturb essential functional and/or structural motifs of the protein.

In this work, the functional consequence of four previously reported point *CYP21A2* mutations - p.R132C, p.R149C, p.M283V, p.E431K- found in Argentine patients is analyzed [12]. We additionally report a novel point mutation in an acceptor splice site in the intron 5 of the gene found in a CCAH patient. Its pathogenic functional implications was assessed by bioinformatic predictions and analyzing the splicing outcome of the gene bearing the c.652-2A>G mutation.

The putative pathogenic mechanisms of the newly described mutations coding for aminoacidic substitution of CYP21A2 were previously analyzed *in silico* by means of mutagenesis modeling and protein stability calculations [12]. In the present work, all the mutant forms showed a lower activity than the WT. In general, the remaining activity of the isolated mutants was reduced to a lesser than 50% of the WT activity with both 17-OHP and progesterone. Accordingly, all the variants predict alleles compatible to the mild non-classical form of the disease. However, in one NCCAH patient one novel point mutation -p.E431K- was found in *cis* with the p.D322G mutation. The highest decrease in enzyme activity was obtained when both mutations were assayed in the same construction, with a residual activity most likely related to the SV form of the disease. Furthermore, the variant p.D322G in isolation revealed a decrease in enzyme activity similar to that described by Bleicken *et al*. [22].

To determine whether the mutations in *CYP21A2* affect protein levels or its activity only, western blot studies were performed with proteins obtained from homogenates of cells transfected with the different constructs. Western blot analysis revealed that the amount of p.R132C, p.R149C and p.M283V mutant proteins is comparable to that of the wild type, suggesting that the reduction in enzymatic activity is not due to a significant decrease in the amount CYP21A2 but rather in the activity itself. For instance, R132 is part of a cluster of basic amino acids suggested to be involved in the redox partner interaction with P_450_ Oxido Reductase (POR) [11]. Mutations in these basic amino acids are likely to cause electrostatic disturbances so an impairment of the enzyme activity by C132 mutation could be expected. These suggestions are supported by the *in silico* modeling results recently published by Haider *et al*. [13] using as template the crystallized bovine CYP21 that shares 79% sequence identity with human CYP21A2. On the other hand, in a previous paper [12] we predicted that the change of a methionine by a cystein in residue 149 could destabilize the protein. Likewise, Haider *et al*. [13] suggested that the presence of p.R149C mutation could disrupt a salt-bridge interaction with E162 and hence a localized destabilization of the tertiary structure of the protein is predicted. Moreover, a recent work illustrated that a p.R149P mutation found in the *CYP21A2* gene showed a residual activity of 16.9% and 23.4% for the conversion of progesterone and 17-OHP, respectively [28]. In addition, the reduction in the enzymatic activity of the mutant p.M283V is in accordance with the positioning of the methionine 283 in the well-conserved helix I, which was suggested to be involved in both heme binding and substrate recognition [11].

The determination of protein levels by western blot in cells transfected with p.E431K, p.D322G and p.E431K plus p.D322G mutations indicate that there is a clear decrease in the amount of protein, suggesting in these mutants either an impaired synthesis or an enhanced degradation of the CYP21A2 protein. A decrease in protein stability and hence in the half-life has been described for p.R483P mutant [29] and a reduction in the amount of protein in the presence of p.D322G mutation was also demonstrated by Bleicken *et al*. [22]. Therefore, the reduction in the enzyme activity in cells transfected with p.E431K and p.D322G could be explained by a decrease in protein expression. Conversely, the synergistic effect determined in enzyme activity when both mutations are located in *cis* is not related to a similar extent reduction in the amount of the protein, suggesting an additional effect in substrate conversion. On the other hand, the results of western blot analysis for p.E431K are in contrast with the prediction that this mutation marginally stabilizes the protein [12]. Since the mutation introduces a lysine, we wonder if a new ubiquitination site was generated. The search for such a consensus motif revealed no obvious ubiquitination site (data not shown).

It is well documented that most of the 21-hydroxilase deficient patients are compound heterozygotes carrying different *CYP21A2* mutations in each allele, being the phenotype of the affected individual dependent on the milder gene defect [10]. Accordingly, based on the almost null residual activity of the protein codified by the c.283-13A/C>G on the homologous allele in one of the NCCAH patients, we previously predicted the p.M283V as a mild mutation [12]. In the present paper, *in vitro* data further confirmed this prediction. Moreover, we recently genotyped a second patient of our cohort showing a NCCAH phenotype as p.M283V/macroconversion (data not shown). However, in the other patients described by Minutolo *et al*. [12] (Table 1), the presence of another mild mutation or the lack of demonstrable mutation on the second allele precluded the prediction of the enzymatic activity of the novel allele. Although the use of approximate models has allowed making some predictions regarding activity with certain accuracy [11]–[13], the magnitude of the putative impairment was not always reliable predicted. Consequently, *in vitro* analyses seem to be necessary to further clarify the severity of a mutated allele for an accurate genetic counseling. Moreover, a second point mutation on the same allele have been described in 21-hydroxylase deficiency and none of the *in silico* models predicted their biological consequences. Indeed, the functional studies performed in this work and in other ones [30]–[33] revealed low enzymatic activities for those alleles with two mild mutations *in cis*.

The presence of a single *CYP21A2* mutated allele has been previously described in patients diagnosed with 21-hydroxylase deficiency [28], [34]–[40]. In fact, in patient 1 and after sequencing the entire gene, the minimal promoter and the regulatory regions, no mutations with the exception of p.R132C were found [12]. Several studies have shown that heterozygous carriers of *CYP21A2* mutations may have an increased risk of hyperandrogenic symptoms and could exhibit ACTH-stimulated 17-OHP values similar to those described in patients with the non-classical form of the disease [41], [42]. Nevertheless, basal and stimulated 17-OHP values in this patient (5.7 and 30 ng/mL, respectively [12]), markedly exceeded the currently cut-off levels used for the diagnosis of NCCAH due to 21-hydroxylase deficiency [16], [43]. Besides, and even though an overall good genotype-phenotype correlation have been reported for this disorder, a recent work by New *et al* [44] described that almost half of the genotypes showed a discordant phenotype. These observations as a whole might indicate that other genetic and/or environmental factors could contribute to modulate the activity of the CYP21A2 enzyme *in vivo* and/or the possible clinical manifestation exhibited by the patients.

In addition, in the present work, we describe a novel acceptor splice mutation in intron 5 in compound heterozygosity with the rare p.R483Q mutation found in a SV patient. *In silico* analysis predicted the disruption of the canonical splicing site by the c.652-2A>G mutation. Besides, two strong putative cryptic splicing sites, one located in the intron 5 and one in exon 6 were predicted. By functional analyses using transient expression of splicing reporter minigenes transfected into HEK-293, HeLa and Y-1 cells, we demonstrate that the allele with the c.652-2A>G mutation completely abolishes the use of the canonical splice site. Instead, in the three cell lines analyzed, the use of the cryptic splicing acceptor site within exon 6 created an mRNA with a 16 nt deletion leading to the appearance of a premature stop codon.

*In vivo,* two possible and non exclusive mechanisms could be related to the pathogenic consequence of c.652-2A>G mutation: the putative formation of a truncated, unstable protein with no enzymatic activity and the mRNA degradation due to a nonsense mediated decay (NMD) mechanism. The *in vitro* approach used in this paper relies on *in vitro* mRNA over-expression from a strong promoter and thus unable to address whether NMD occurs *in vivo* and its relevance on the observed pathogenic phenotype. Nevertheless, and regardless of the underlying mechanism, a severe allele is strongly suggested by the substitution in the intron 5 acceptor splice site.

It should be noted that although mutations in the consensus splicing acceptor or donor sites of the *CYP21A2* gene have been previously reported in 21-hydroxylase deficient patients [19], [45]–[48], functional consequences have only been addressed for the c.282+1G>A (IVS2+1G>A) mutation [49]. Furthermore, the strategy used in this paper allowed for the first time, the demonstration of the consequence of a splice mutation also in a steroidogenic cell line. Similarly to our finding, the c.282+1G>A mutation revealed the use of a cryptic splicing site in the *CYP21A2* gene. Differently to the mutation described here, two types of transcripts from this mutant *CYP21A2* gene were reported.

Although the mutation in the intron 5 acceptor splicing site predicts a severe allele, the patient herein described disclosed a phenotype most likely related to the previously reported *in vitro* enzymatic activity of the rare p.R483Q allele [50], [51]. To our knowledge, only three other patients with the p.R483Q mutation were reported: one boy diagnosed as NCCAH [52] and two girls with clinical symptoms and hormonal values compatible with the classical SV form [50], [51]. Considering that the distinction between SV and NCCAH is more difficult in males, the patient herein described supports the evidence that the p.R483Q mutation might be related to the SV form of the disease rather than to the non-classical one.

In conclusion, we described the functional consequences of four previously described mutations and a novel one found in 21-hydroxylase-deficient patients from the Argentinean population. All the isolated mutants coding for aminoacidic substitution of CYP21A2 predict mild alleles related to the NCCAH form, while the presence of two mild mutations on the same allele predicts a classical SV one. The c.652-2A>G mutation is herein described for the first time. We found that the allele with this acceptor splice site mutation completely abolishes the use of the canonical splice site. Although bioinformatic tools predicted the putative use of two different cryptic splicing sites, functional analyses revealed the use of only the cryptic splice acceptor site within exon 6. A severe allele is strongly suggested due to the appearance of a premature stop codon.

## Supporting Information

Text S1 Clinical characteristics and hormonal profile of the patient in whom the novel mutation in the acceptor splicing site was found.
(DOCX)
