# Supplementary material for: Functional Studies of p.R132C, p.R149C, p.M283V, p.E431K, and a Novel c.652-2A>G Mutations of the CYP21A2 Gene
Source: PLoS One. 2014 Mar 25;9(3):e92181. doi: 10.1371/journal.pone.0092181 (PMC3965420; doi:10.1371/journal.pone.0092181)
Supplement: Text S1 — Clinical characteristics and hormonal profile of the patient in whom the novel mutation in the acceptor splicing site was found. (DOCX) [file pone.0092181.s001.docx]

The patient, a 31 year-old woman, was referred to the adult unit to reevaluate the diagnosis of CAH. She was the first child born from healthy non-consanguineous parents of Portuguese origin. She had been referred to a pediatric unit at the age of 6 months due to clitoromegaly, advanced bone age (2 years) and pubic hair. She was diagnosed as 21-hydroxylase deficiency CAH and treated with prednisone until the age of 8 when she stopped treatment and medical assistance on her mother account. During childhood she was always taller than the average, and at the age of 10, she achieved final height. She never had surgical correction of her genitalia.

At the age of 12 she was recalled to the Paediatric Endocrine Unit. The patient was treated with hydrocortisone until she was 15 years old. During treatment, she presented breast development followed by menarche. At the age of 18, she stopped treatment and missed medical follow-up.

Although she had not received any treatment for almost 13 years, she continues having 3-4 days menstruation periods each 25-40 days. At the time of the interview, she complained of hirsutism but she never had hair growth in the chin or in the face. She never became pregnant in spite of never using contraceptive methods. Physical examination disclosed a height of 159 cm, 0.1 SDS (target height: 164 ± 8 cm), a BMI of 23 kg/m², well developed breasts and typical female distribution of body fat. She was moderately hirsute (Ferriman score 13). There was not sexual ambiguity of the external genitalia except for a mild clitoromegaly (2 cm) without labial fusion and normal urethral orifice and vaginal opening (Prader I). Laboratory data during follicular phase showed high hormonal serum levels compatible with values in the range of classical adrenal hyperplasia: basal 17-hydroxy-progesterone 300 ng/mL, post ACTH test 1050 ng/mL, androstenedione 16 ng/mL, and testosterone 2.1 ng/ml. Levels of cortisol were not increased after ACTH stimulation. Treatment was started with prednisone 4 mg/day and switched to 5 mg/day. Five months later she became pregnant and a healthy girl was born.
